# Effects of *Boswellia Serrata* and Whey Protein Powders on Physicochemical Properties of Pork Patties

**DOI:** 10.3390/foods9030334

**Published:** 2020-03-12

**Authors:** Fengqi Yang, Won-Young Cho, Nayeon Lee, Da-Hee Kim, Jihye Lee, Hyun-Jin Lee, Han Geuk Seo, Chi-Ho Lee

**Affiliations:** Department of Food Science and Biotechnology of Animal Resources, Konkuk University, Seoul 05029, Korea; yfq426@naver.com (F.Y.);

**Keywords:** *Boswellia serrata*, whey protein, sensory evaluation, texture, antioxidant, functional food

## Abstract

Processed meat products are prone to oxidative damage and quality decline during storage; however, these problems can be mitigated by the proper formulation of meat productions. This study evaluated the effects of natural anti-oxidants found in *Boswellia serrata* (B), whey protein powder (W), and their combination on pork patties during storage, exploring changes in textural properties and lipid oxidation susceptibility. The 2% whey-added group exhibited a higher crude protein content than the untreated control group. The highest water-holding capacity and lowest cooking losses were observed in mixed-additive groups (WB_1_ (2% W/0.5% B) and WB_2_ (2% W/1.0% B), and the highest sensory scores for overall acceptability were obtained for WB_1_. Adding *B. serrata* can neutralize the hardness caused by whey powder, thereby improving palatability. From 7 d (days 7), the extents of lipid oxidation, determined using 2-thiobarbituric acid-reactive substances (TBARS) analysis, for the WB_1_ and WB_2_ groups were significantly lower than that of the control group. The WB_1_ and WB_2_ groups exhibited substantially suppressed total bacterial colony and *Escherichia coli* counts relative to the control group. Our findings suggest that the additive combination of *B. serrata* and whey protein powders can suppress lipid oxidation, improve storage stability, and enhance textural properties in the production of functional pork patties.

## 1. Introduction

Lipid oxidation is a principal factor affecting the quality and acceptability of meat products. Ground meat, in particular, is especially vulnerable to microbial contamination and lipid oxidation during processing and storage [1,2]. Such processes can produce off-flavors and toxic degradants, negatively affect sensory properties, lead to the loss of bioactive compounds and nutritional value, degrade color, and reduce shelf life and economic returns [3,4,5]. Uncontrolled oxidation and microbial contamination have now become one of the biggest economic problems in the processed meat industry [2].

*Boswellia serrata* and whey protein are natural substances that have antioxidant properties [1,6]. Consumers generally prefer natural antioxidants [7,8] over synthetic antioxidants such as butylated hydroxyanisole (BHA), butylated hydroxytoluene (BHT), and tertiary-butylhydroquinone (TBHQ), considering the side effects of synthetic antioxidants. Furthermore, maintaining moisture, lipids, and food composition as well as improving flavor are necessary components in the applications of meat products. Non-meat protein additives such as whey protein are added to ground pork products primarily to increase product yield, lower production cost, boost water-holding capacity (to enhance juiciness), and improve the textural and functional properties of the meat product [9,10,11].

*Boswellia serrata* is an important traditional medicinal plant grown in India, Northern Africa, and the Middle East [12,13]. Commonly known as Indian frankincense, the oleo-gum resin obtained from *B. serrata* possesses antioxidant, anti-inflammatory, antimicrobial, anticancer, and analgesic properties [14,15,16]. The main phytochemical components of the *B. serrata* resin are essential oils, gum, and resin, as well as various functional compounds such as terpenoids, β-boswellic acid, phenolic compounds, and polysaccharides [12,17,18]. Whey is a major by-product of cheese-making and has long been considered as waste [19]. It is composed of β-lactoglobulin, α-lactalbumin, immunoglobulin, bovine serum albumin, lactoferrin, lactoperoxidase, glycomacropeptide, and growth factors, as well as numerous biologically active factors and enzymes [20,21]. Because whey protein is a high-quality protein with an amino acid content and rapid digestibility that are biologically superior to most other proteins, much attention has been focused on it [22]. Recently, a number of studies have shown that whey protein and its related components have biological functions with specific health benefits, including antioxidant activity, immune regulation, and antibacterial and anticancer properties [23].

Although the effects of adding whey powder, tocopherol, spices, and phenolic compounds sourced from plant extracts on the functional properties of meat products have been studied [24,25,26,27], no researchers have considered the textural properties and antioxidation behaviors of pork patties supplemented with combinations of *B. serrata* and whey powders. This study aims to determine the effects of mixed *B. serrata* and whey powders on the quality characteristics of pork patties, including their impact on shelf life and oxidative stability.

## 2. Materials and Methods

### 2.1. Formulation and Preparation of Pork Patties

Pork patties containing *B. serrata* and whey powders were formulated as shown in Table 1. The *B. serrata* and whey powders were obtained from a local market and ESfood (Gyeonggi-do, Korea), respectively. A pork fillet and back fat were purchased from a local market (Seoul, Korea). All visible connective tissue and fat were removed from the pork fillet with a butcher’s knife. The lean pork fillet and back fat were ground through a 5 mm plate, divided into five equal portions, and then mixed with the appropriate amounts of *B. serrata* and whey powders for 12 min using a stand mixer (SM 246, Poking Industrial Co., Ltd., Hong Kong, China). After thorough mixing, the pork mixtures were formed into identically sized patties (100 ± 1 g each) using a rectangular burger press (Spikomat Ltd., Nottingham, UK). The pork patties were vacuum packaged and stored at 4 °C for 0, 7, 14, or 21 d while the experiments were carried out in triplicate for each pork patty formulation.

### 2.2. Proximate Composition

The moisture, ash, and crude protein and fat contents of the pork patties were analyzed using official methods (AOAC, 2012) [28]. The crude protein and crude fat contents were respectively determined by the Kjeldahl and Soxhlet methods. The moisture content was determined at 105 °C using a drying oven and the crude ash content was measured at 550 °C via the dry ashing method.

### 2.3. pH and Color

Following the homogenization of the pork patty samples (2 g) with distilled water (18 mL) for 60 s at 3220 × *g* with a homogenizer (AM-1, Nihon Seiki Kaisha Co., Ltd., Nagoya, Japan), their pH values were measured with a pH meter (LAQUA F-71, Horiba Co., Kyoto, Japan).

The surface color of each uncooked pork patty was determined using a colorimeter (Minolta Chroma Meter CR-210, Japan) on day 0 and during specified storage intervals, measuring the CIE L^*^ (lightness), CIE a^*^ (redness), and CIE b^*^ (yellowness) values. The colorimeter was calibrated with a white standard plate (CIE L^*^ = +97.83, CIE a^*^ = −0.43, and CIE b^*^ = +1.98).

### 2.4. Water-Holding Capacity (WHC) and Cooking Loss

The water-holding capacity of each patty was determined according to the method of Akwetey and Yamoah [9]. A sample (5 g) was mixed thoroughly with distilled water (10 mL) in a tube and the mixture was centrifuged at 2000 rpm for 15 min at 15 °C. Then, the supernatant was carefully removed and the remaining sample was weighed. The WHC (%) was calculated as follows:

WHC (%) = (weight of sample after removing supernatant/weight of sample mixed with distilled water) × 100

The raw weight of each sample was obtained before and after cooking to an internal temperature of 75 °C; the patties were allowed cool at room temperature prior to weighing and texture profiling. The cooking loss from the patty samples was computed using the following equation by Murphy et al. [29]:

Cooking loss (%) = (raw patty weight−cooked patty weight) × 100(1)

### 2.5. Texture Profile Analysis (TPA)

The samples used in the cooking loss analysis were subjected to TPA (three replicates) using a texture analyzer (CT3-1000, Brookfield Engineering Laboratories, Inc., Middleboro, MA, USA). Samples were cut into 20 × 20 × 10 mm^3^ (length × width × height) portions. The operating conditions for the texture analyzer were set as follows: distance = 8.0 mm, pre-test speed = 5.0 mm/s, post-test speed = 2.0 mm/s, test speed = 2.0 mm/s, and force = 5.0 g. Textural attributes such as hardness (the peak force generated by the product on first compression (kg)), springiness (the ration of storage deformation to total deformation in the second loading cycle in texture profile analysis), cohesiveness (the ratio of active work to total work in the second loading cycle in the TPA), gumminess (hardness × cohesiveness (kg)), and chewiness (hardness × cohesiveness × springiness (kg)) were analyzed.

### 2.6. 2-Thiobarbituric Acid-Reactive Substances (TBARS)

Lipid oxidation was determined using the 2-thiobarbituric acid reactive substances (TBARS) method described by Buege and Aust [30]. Each sample (5 g) was homogenized with distilled water (15 mL) and BHT (100 μL, 6% in 100% ethanol) at 13,000 rpm for 1 min. After homogenization, a solution aliquot (2 mL) was transferred into a test tube, mixed with a trichloroacetic acid / 2-thiobarbituric acid reagent (4 mL), and heated in an 80 °C water bath for 15 min. After cooling in cold water, the mixture was centrifuged at 2000× *g* for 10 min at 25 °C, and then filtered through Whatman paper No. 4. Sample absorbance was measured at 531 nm using a spectrophotometer (UV/Vis Spectrophotometer, Mecasys, Daejeon, Korea). Results were expressed as 2-thiobarbituric acid-reactive substances in mg malonaldehyde (MDA)/kg meat. A standard curve was prepared using 1,1,3,3-tetraethoxypropane (TEP) for calculations.

### 2.7. Volatile Basic Nitrogen (VBN)

The VBN contents of the samples were measured by Conway’s microdiffusion method with a slight improvement [31]. A sample (5 g) was mixed with distilled water (15 mL) and homogenized at 10,000 rpm for 60 s. The homogenate was filtering and placed in the Conway’s unit. The unit was then sealed and slowly agitated after added the Conway reagent. Titrated with 0.02 N sulfuric acid after incubation at 37 °C for 120 min.

### 2.8. Microbiological Analysis

Microbiological analysis of the pork patties was performed using 3M Petrifilm (3M, St. Paul, MN, USA). Patty samples (25 g) were homogenized with a 0.85% sterile saline solution (225 mL) in a side-filter bag for 90 s using a bag mixer (Bagmixer 400 W; Interscience, Woburn, MA, USA). The aerobic count plates were incubated at 37 ± 1 °C for 48 ± 2 h to determine the total bacterial counts. Coliform and *Escherichia coli*/coliform count plates were incubated at 37 ± 1 °C for 24–48 h to determine the *E. coli* and total coliforms. The colonies were counted, and results were expressed as log colony forming units per gram sample (CFU/g).

### 2.9. Sensory Evaluation

Sensory tests of the patties with or without the *B. serrata* and whey powders were performed at the Department of Food Science and Biotechnology of Animal Resources, Konkuk University. Panels for the sensory evaluation consisted of 20 randomly assigned trained researchers (8 men and 12 women with an average age of 27.8 years). Samples were cut into blocks (1 × 1 × 1 cm^3^), placed on a white food plate at room temperature, and then served to the panelists at random. After eating one sample, panelists were asked to gargle with water and eat another sample one or two minutes later. The color (1 = undesirable, 9 = desirable), flavor (1 = undesirable, 9 = desirable), juiciness (1 = dry, 9 = juicy), tenderness (1 = tough, 9 = tender), and overall acceptability (1 = undesirable, 9 = desirable) of the cooked samples were evaluated using a nine-point hedonic scale.

### 2.10. Statistical Analysis

A total of 300 samples were analyzed ((20 patties × 5 batches × 3 (triplicate manufacture)) using SPSS 24.0 software (SPSS Inc., Chicago, IL, USA) using one-way analyses of variance and Tukey’s test. *p*-values of less than 0.05 were considered significant.

## 3. Results and Discussion

### 3.1. Proximate Analysis, WHC, and Cooking Loss

The results of the proximate composition measurements, WHC, and cooking losses from the pork patties after adding the *B. serrata* and whey powders are shown in Table 2. The crude fat and ash contents of the pork patties do not change significantly with the addition of either powder or their combination. Adding *B. serrata* powder to the patties generates no difference from the control group in terms of moisture and crude protein contents, but the moisture content is significantly decreased with the addition of whey powder (*p* < 0.05). This may be because of the reduction in moisture content owing to the increase of dry matter in the formulation. A similar result was reported by Serdaroglu [32], in which the addition of whey powder reduced the moisture content in beef meatballs. Furthermore, the crude protein contents in all treatment groups containing whey powder exhibit significantly higher values than the control group (*p* < 0.05). This result is expected because whey powder is rich in protein, and its addition results in increased protein content, as was similarly described for chicken breast meat injected with whey protein [33].

Cooking losses can influence both sensory properties and product quality. From Table 2, the highest WHC is observed for the WB_1_ and WB_2_ groups (*p* < 0.05) followed by the W and B samples, whereas the CON group has the lowest WHC. Furthermore, cooking losses are significantly lower for WB_1_ and WB_2_ than for the control group (*p* < 0.05), which reaffirms the water retention capabilities of the *B. serrata* and whey powders. Taylor and Walsh [34] reported that textured whey protein significantly increased water retention, and El-Magoli et al. [35] showed that the WHC depends on water-protein interactions. In agreement with those results, we can conclude that powders of *B. serrata* and whey have the ability to retain moisture and prevent patties from drying out, thereby improving palatability.

### 3.2. pH Analysis

**Figure 1:**
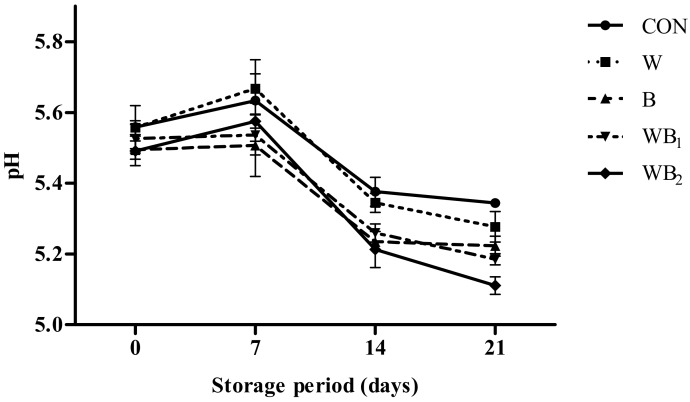
Changes in the pH values of pork patties with different treatments during refrigerated storage at 4 °C for 21 d. Error bars indicate standard deviations. CON (●), control (no additive(s)); W (■), addition of 2% whey powder; B (▲), addition of 0.5% *B. serrata* powder; WB_1_ (▼), addition of 2% whey powder and 0.5% *B. serrata* powder; WB_2_ (◆), addition of 2% whey powder and 1% *B. serrata* powder.

The pH values of the uncooked pork patties during storage are presented in Figure 1. pH is a major factor affecting the quality of processed meat products. Changes in pH can affect properties such as freshness, texture, and color [36]. The pH values of all samples exhibit a slight upward trend with time until 7 days, which may be due to the growth of microorganisms and the enzymatic breakdown of proteins to produce alkaline substances [37]. During storage, the pH values of the W and CON groups are nearly identical, and no significant differences between groups B, WB_1_, and WB_2_ are observed until 14 days. However, after 7 days, the pH values in the *B. serrata* powder treatment groups (B, WB_1_, and WB_2_) are significantly lower compared to those in the control group (*p* < 0.05). Clearly, this change is mainly caused by the added *B. serrata* powder; specifically, it most likely results from the introduction of acidic components such as 11-keto-β-acetyl-β-boswellic acid (KBA), acetyl-11-keto-β-boswellic acid (AKBA), and acetyl-α-boswellic acid (AαBA) into the pork patties [17]. Similar decreasing pH trends have been observed upon the addition of edible seaweed that contains alginic acid to meat products [38]. As acids can effectively inhibit the growth of microorganisms, these findings could improve the storability of pork patties through the addition of *B. serrata*.

### 3.3. Color Measurements

Results obtained from the color measurements of the pork patties are presented in Table 3. The color of meat, which is associated with freshness and pH, will change over time depending on the concentrations of deoxymyoglobin, metmyoglobin, and oxymyoglobin [39]. Compared with the fresh patties (0 day), the L^*^ values of the 21 days samples decrease significantly for all treatment groups, and the a^*^ readings exhibit significant reductions in all groups from 7 to 14 days (*p* < 0.05). These changes originate from the metmyoglobin that is generated by pigment oxidation and reduces the lightness and redness values. Moreover, when the pH is low, the meat also becomes pale. The L^*^ values are higher for the B group than for CON at 0 and 14 days; furthermore, the L^*^ values are higher in WB_2_ than in CON at 14 days (*p* < 0.05). For other storage periods, all treatment groups are lighter (*p* < 0.05) than the control group, which could be due to the prevention of pigment oxidation by the added *B. serrata* and whey powders. Similarly, previous reports have indicated that myoglobin oxidation and lipid oxidation are interrelated and that they affect the color of meat [40]. Our results agree with those obtained by Atughonu et al. [41], who found that non-meat proteins led to a higher value of L^*^ owing to their diluent effects on myoglobin pigments. The a^*^ values of CON significantly decrease (*p* < 0.05) from 14 to 21 days, but all treated groups’ values remain stable. Similar results were observed by Zhang et al. and Ozer et al. [42,43]. Lipids change color from white to yellow as a result of oxidation. In most samples, the b^*^ values are similar at the beginning and end of storage. However, the three treatment groups, namely, WB_1_, WB_2_, and W display lower b^*^ values (*p* < 0.05) than the control group at 7 and 21 days, respectively. These results indicate that the additives employed in this study exhibit a protective effect with respect to discoloration of the meat product.

### 3.4. Texture Profile Analysis (TPA)

Table 4 shows the effects of the *B. serrata* and whey powders on the textural properties of cooked pork patties, in terms of attributes such as springiness, cohesiveness, chewiness, gumminess, and hardness. Pork patties with added whey powder exhibit higher (*p* < 0.05) values with regard to chewiness, gumminess, and hardness than the pork patties in the control group. No variations in springiness and cohesiveness are observed among the test and control groups. Lower values of hardness are observed in the B, WB_1_, and WB_2_ groups as compared to the CON and W groups (*p* < 0.05). Furthermore, owing to the reduced hardness, *B. serrata* treatment alone (group B) results in slightly lower chewiness in comparison to the WB_1_, CON, and W groups (*p* < 0.05). Adding *B. serrata* can neutralize the hardness caused by whey powder, thereby improving palatability. Similar changes in texture profiles that increased the hardness and chewiness but did not affect the springiness and cohesiveness with added whey have been reported by Andic et al. [11]; furthermore, such changes were attributed to the β-lactoglobulin contained in whey powder. This substance has good thermogelation characteristics and can be denatured when heated in a usually thermally irreversible manner. The reduction in the hardness value can probably be attributed to the moisture-retention properties of the *B. serrata* powder. This is consistent with the results obtained by Wan Rosli et al. [44], who reported that addition of oyster mushroom results in higher water retention that contribute to hardness reduction in chicken patties. In addition, Verma et al. [45] found that, owing to its moisture-retention properties, sweet potato powder also reduced hardness values in low-fat formulated pork patties.

### 3.5. Storage Stability

**Figure 2:**
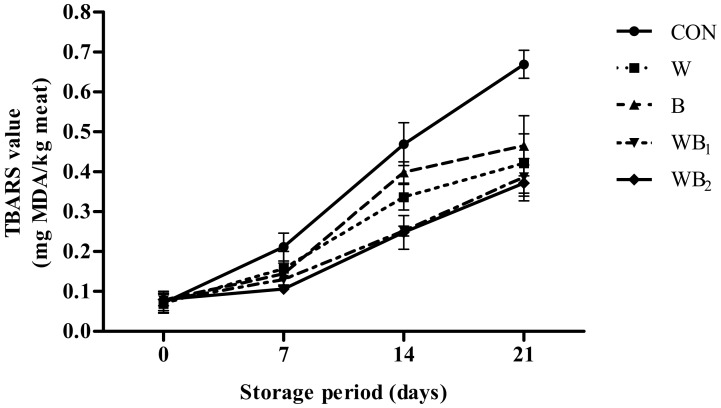
Changes in the 2-thiobarbituric acid reactive substances (TBARS) values of pork patties with different treatments during refrigerated storage at 4 °C for 21 days. Error bars indicate standard deviations. CON (●), control (no additive(s)); W (■), addition of 2% whey powder; B (▲), addition of 0.5% *B. serrata* powder; WB_1_ (▼), addition of 2% whey powder and 0.5% *B. serrata* powder; WB_2_ (◆), addition of 2% whey powder and 1% *B. serrata* powder.

The changes in the TBARS values during the storage of pork patties are shown in Figure 2. Although the values for the control group increase continuously over 21 days (*p* < 0.05), the other treatment groups exhibit no significant differences between 0 and 7 days, after which their TBARS values being to rise. From 7 days, the TBARS values of the WB_1_ and WB_2_ groups are significantly lower than that of the control, and the TBARS values of the WB_2_ sample, in particular, are the lowest (*p* < 0.05). The detectable threshold of oxidized flavor in meat is observed when TBRAS values are 0.5–2.0 mg MDA/kg [46]. At day 14, the TBARS value of the CON group is 0.47, however the treatment groups are still lower than 0.5 until 21 days. According to other reports [17,23,47], this could be due to the addition of *B. serrata*, which contains terpenoids (KBA, AKBA, and AαBA), phenolic compounds, diterpene alcohols as well as the addition of whey, which can prevent lipid oxidation. Although there are no reports on meat products showing that *Boswellia* species display antioxidation effects, similar research has stated that rosemary contains phenol diterpenes (carnosic acid and rosmarinic acid) with antioxidant properties [48].

**Figure 3:**
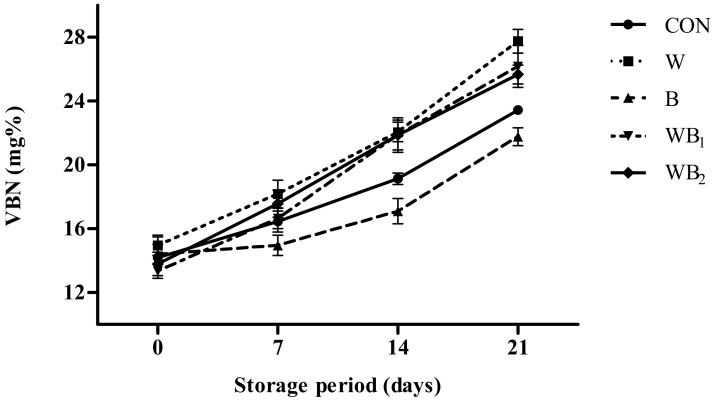
Changes in the volatile basic nitrogen (VBN) values of pork patties with different treatments during storage. Error bars indicate standard deviations. CON (●), control (no additive(s)); W (■), addition of 2% whey powder; B (▲), addition of 0.5% *B. serrata* powder; WB_1_ (▼), addition of 2% whey powder and 0.5% *B. serrata* powder; WB_2_ (◆), addition of 2% whey powder and 1% *B. serrata* powder.

The VBN content of the pork patties tends to increase with storage time, with the B group showing the lowest (*p* < 0.05) value during days 7–21 (Figure 3). The VBN contents of the B and CON samples are significantly lower than those of the other groups that contain whey powder after 14 days. According to Korea Food Law (2002), the limit of allowable VBN content is 20 mg/100 g or less in meat. The value of CON group is close to 20 mg% after about 14 days of storage, however the B group take approximately 21 days to reach the same value. This suggests that the addition of *B. serrata* may delay protein degradation, possibly owing to the antibacterial effects of the additive [47]. From our results, it is observed that samples treated with whey powder exhibit a higher VBN value compared to those in CON. During storage, meat products decompose, with proteins being degraded into amino acids and producing low molecular weight inorganic nitrogen. Therefore, it is speculated that an increase in the nitrogen content with the addition of whey powder results in the higher VBN values. These results agree with those obtained by Ha et al. [33], who found that the injection of whey protein in chicken breast meat produced higher VBN values compared to those of the control group.

**Figure 4:**
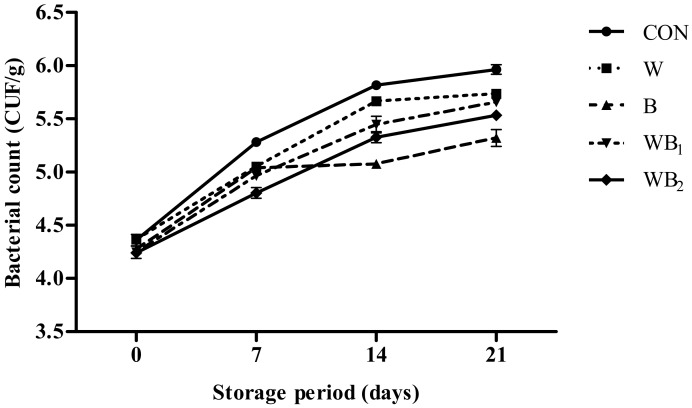
Effect of *B. serrata* and whey powders on the number of aerobic bacteria in pork patties during storage. Error bars indicate standard deviations. CON (●), control (no additive(s)); W (■), addition of 2% whey powder; B (▲), addition of 0.5% *B. serrata* powder; WB_1_ (▼), addition of 2% whey powder and 0.5% *B. serrata* powder; WB_2_ (◆), addition of 2% whey powder and 1% *B. serrata* powder.

The results of microbiological analyses of the pork patties during storage are shown in Figure 4. The total aerobic bacterial counts in all groups increase with storage time (*p* < 0.05). Initially, no significant differences are observed among the treatments containing *B. serrata*, although their microbe counts are slightly lower than those in W and CON. The rich terpenoid content in *B. serrata* may be an important factor with regard to its antibacterial activity [49]. The control group exhibits the highest bacterial counts from 7 to 21 days. The number of aerobic counts is significantly lower in WB_2_ compared to the other treatments during 0–7 days; however, the B group shows the slowest growth rate and the lowest total bacterial count values from 14 days (*p* < 0.05). It may be that the AKBA in *B. serrata* can inhibit the formation of biofilms and lactoferrin in whey powder, thereby slowing bacterial growth, owing to their antibacterial capabilities [12,50]. Therefore, *B. serrata* and whey protein are capable of maintaining stability during storage, and *Boswellia* exhibits better antibacterial activity. Finally, neither *E. coli* nor other coliform bacteria were detected in any sample during storage in this study (data not shown).

### 3.6. Sensory Evaluation

**Figure 5:**
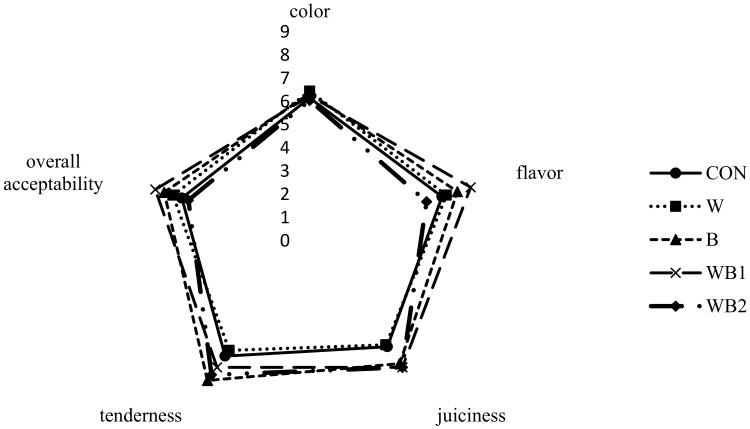
Effect of *Boswellia serrata* and whey powders on the sensory evaluation of pork patties. CON (●), control (no additive(s)); W (■), addition of 2% whey powder; B (▲), addition of 0.5% *B. serrata* powder; WB_1_ (×), addition of 2% whey powder and 0.5% *B. serrata* powder; WB_2_ (◆), addition of 2% whey powder and 1% *B. serrata* powder.

Figure 5 shows the sensory evaluation of the differently treated pork patties. All products achieve similar color scores irrespective of the formulation. Compared to other groups, the WB_1_ sample results in the highest sensory scores with regard to flavor, juiciness, and overall pork patty acceptability. This may be due to the aromatic components in *Boswellia*, which mainly comprise α-pinene, β-myrcene, linalool, and sesquiterpenoids that can convey a pleasant odor [47]. However, when added at 1%, the aroma, similar to spices, is too strong, such that WB_2_ has the lowest score for flavor (although without a significant difference compared to the control and whey groups). The scores for juiciness and tenderness are not significantly different among the WB_1_, WB_2_, and B samples; however, they are significantly higher than those of the W group (*p* < 0.05). The lower cooking losses are a result of the greater water-holding capacity arising from the *Boswellia* in WB_1_, WB_2_, and B; therefore, higher juiciness and tenderness values are observed, consistent with the TPA results.

## 4. Conclusions

This study shows that combined whey protein and *B. serrata* powders can extend the shelf life of pork patties by inhibiting lipid oxidation, reducing pH and bacterial growth, and effectively improving quality characteristics. Introducing additives into pork patties reduces the meat content and cost compared to ordinary patties. Furthermore, *B. serrata* and whey protein are natural antioxidants and can protect the product from discoloration and improve taste, which can increase consumer satisfaction. Further studies can apply these benefits to fermented meat products.

## Figures and Tables

**Table 1:** Formulation (wt%) of pork patties containing *Boswellia serrata* and whey powders.

Ingredients	Treatment ^1^				
	CON	W	B	WB_1_	WB_2_
Pork fillet	75	75	75	75	75
Back fat	25	25	25	25	25
Salt	1	1	1	1	1
Whey	0	2	0	2	2
*B. serrata*	0	0	0.5	0.5	1
Total	101	103	101.5	103.5	104

^1^ CON, control (no additive(s)); W, addition of 2% whey powder; B, addition of 0.5% *B. serrata* powder; WB_1_, addition of 2% whey powder and 0.5% *B. serrata* powder; WB_2_, addition of 2% whey powder and 1% *B. serrata* powder.

**Table 2:** Proximate composition (%) analysis, WHC (%), cooking loss (%) of pork patties formulated with *Boswellia serrata* and whey powders.

Item ^1^	CON	W	B	WB_1_	WB_2_	SEM ^2^	*p*-Value
Ash	1.84 ± 0.01	1.90 ± 0.07	1.83 ± 0.02	1.90 ± 0.05	1.91 ± 0.02	0.03	0.14
Moisture	54.50 ± 0.37 ^a^	53.09 ± 0.24 ^b^	53.50 ± 0.37 ^a,b^	53.02 ± 0.80 ^b^	52.52 ± 0.20 ^b^	0.37	*
Crude fat	26.02 ± 0.86	25.31 ± 0.44	25.63 ± 0.69	25.25 ± 0.35	25.13 ± 0.15	0.45	0.35
Crude protein	17.15 ± 0.85 ^b^	18.89 ± 0.71 ^a^	17.12 ± 0.36 ^b^	18.79 ± 0.75 ^a,b^	18.62 ± 0.36 ^a,b^	0.52	*
WHC ^3^	46.73 ± 0.27 ^d^	48.35 ± 0.62 ^b,c^	47.76 ± 0.07 ^c^	48.82 ± 0.29 ^a,b^	49.26 ± 0.10 ^a^	0.27	***
Cooking loss	42.54 ± 0.31 ^a^	39.80 ± 0.86 ^b^	39.11 ± 0.69 ^b^	38.53 ± 0.44 ^b,c^	37.71 ± 0.71 ^c^	0.45	***

^1^ CON, control (no additive(s)); W, addition of 2% whey powder; B, addition of 0.5% *B. serrata* powder; WB_1_, addition of 2% whey powder and 0.5% *B. serrata* powder; WB_2_, addition of 2% whey powder and 1% *B. serrata* powder. ^2^ SEM: standard error of the mean. ^a–^^d^ Means within a row with different letters are significantly different (*p* < 0.05). * *p* < 0.05; ***p < 0.001. ^3^ WHC: water holding capacity

**Table 3:** Effects of *Boswellia serrata* and whey powders on the color of pork patties during storage.

L* (Lightness)	**Treatment ^1^**						***p*-Value**	
**Storage (Days)**	**CON**	**W**	**B**	**WB_1_**	**WB_2_**	**SEM ^2^**	**Treatment**	**Storage**
0	65.32 ± 0.30 ^bA^	65.55 ± 0.30 ^bA^	66.85 ± 0.25 ^aA^	65.11 ± 0.38 ^bAB^	65.34 ± 0.43 ^bA^	0.24	***	***
7	64.29 ± 0.87 ^bAB^	65.38 ± 0.22 ^aA^	65.76 ± 0.17 ^aB^	65.34 ± 0.30 ^aA^	65.16 ± 0.33 ^abAB^	0.32	**	**
14	63.45 ± 0.17 ^cBC^	64.11 ± 0.58 ^bcB^	66.38 ± 0.56 ^aAB^	64.23 ± 0.51 ^bcBC^	64.81 ± 0.38 ^abAB^	0.33	**	***
21	62.98 ± 0.44 ^cC^	64.25 ± 0.56 ^bB^	65.63 ± 0.49 ^aB^	64.10 ± 0.63 ^bC^	64.50 ± 0.12 ^bB^	0.34	**	***
							*	
*A* * (redness)	CON	W	B	WB_1_	WB_2_			
**Storage (days)**								
0	12.86 ± 0.18 ^aA^	11.97 ± 0.52 ^abA^	11.44 ± 0.47 ^bA^	11.89 ± 0.32 ^bA^	11.94 ± 0.49 ^bA^	0.29	***	**
7	12.56 ± 0.13 ^aA^	11.79 ± 0.26 ^bA^	11.09 ± 0.15 ^cA^	11.83 ± 0.03 ^bA^	11.91 ± 0.24 ^bA^	0.13		***
14	10.45 ± 0.39 ^aB^	10.40 ± 0.15 ^aB^	8.75 ± 0.11 ^bB^	10.10 ± 0.38 ^aB^	10.29 ± 0.25 ^aB^	0.20		***
21	9.57 ± 0.11 ^aC^	9.80 ± 0.36 ^aB^	8.74 ± 0.13 ^bB^	9.90 ± 0.18 ^aB^	9.99 ± 0.36 ^aB^	0.18		***
							
*B* * (yellowness)	CON	W	B	WB_1_	WB_2_			
**Storage (days)**								
0	7.75 ± 0.24	7.61 ± 0.13	7.52 ± 0.17	7.46 ± 0.21	7.43 ± 0.06 ^A,B^	0.12	***	0.112
7	7.74 ± 0.05 ^a^	7.54 ± 0.40 ^ab^	7.75 ± 0.24 ^a^	7.45 ± 0.15 ^a,b^	7.20 ± 0.10 ^b,B^	0.16	0.440	*
14	7.60 ± 0.07	7.48 ± 0.05	7.56 ± 0.22	7.42 ± 0.09	7.64 ± 0.07 ^A^	0.08	0.395	0.098
21	7.79 ± 0.08 ^a^	7.36 ± 0.13 ^b^	7.59 ± 0.12 ^ab^	7.41 ± 0.16 b	7.30 ± 0.24 ^bB^	0.11	0.960	**
							**	

^1^ CON, control (no additive(s)); W, addition of 2% whey powder; B, addition of 0.5% *B. serrata* powder; WB_1_, addition of 2% whey powder and 0.5% *B. serrata* powder; WB_2_, addition of 2% whey powder and 1% *B. serrata* powder. ^2^ SEM: standard error of the mean. ^a–c^ Means within a row with different letters are significantly different (*p* < 0.05). ^A–C^ Means within a column with different letters are significantly different (*p* < 0.05). * *p* < 0.05; ** *p* < 0.01; *** *p* < 0.001.

**Table 4:** Texture profile analysis of pork patties formulated with *Boswellia serrata* and whey powders ^1.^

Property	CON	W	B	WB_1_	WB_2_	SEM ^2^	*p*-Value
Springiness (mm)	0.75 ± 0.02	0.75 ± 0.02	0.74 ± 0.02	0.75 ± 0.01	0.74 ± 0.01	0.01	0.197
Cohesiveness	0.21 ± 0.02	0.21 ± 0.02	0.23 ± 0.03	0.24 ± 0.04	0.24 ± 0.02	0.01	0.058
Chewiness (kg)	3.29 ± 0.08 ^b^	3.49 ± 0.08 ^a^	3.11 ± 0.09 ^c^	3.26 ± 0.06 ^b^	3.21 ± 0.13 ^b,c^	0.04	***
Gumminess (kg)	4.39 ± 0.06 ^b^	4.68 ± 0.09 ^a^	4.21 ± 0.30 ^b^	4.36 ± 0.04 ^b^	4.36 ± 0.13 ^b^	0.07	***
Hardness (kg)	20.53 ± 0.79 ^b^	21.87 ± 0.54 ^a^	18.07 ± 0.60 ^c^	18.49 ± 0.55 ^c^	18.19 ± 0.50 ^c^	0.27	***

^1^ CON, control (no addition); W, addition of 2% whey powder; B, addition of 0.5% *B. serrata* powder; WB_1_, addition of 2% whey powder and 0.5% *B. serrata* powder; WB_2_, addition of 2% whey powder and 1% *B. serrata* powder. ^2^ SEM: standard error of the mean. ^a–^^d^ Means within a row with different letters are significantly different (*p* < 0.05). *** *p* < 0.001

## References

[1] Ulu H. (2004). Effect of wheat flour, whey protein concentrate and soya protein isolate on oxidative processes and textural properties of cooked meatballs. Food Chem..

[2] Bai J.J., Lee J.G., Lee S.Y., Kim S., Choi M.J., Cho Y. (2017). Changes in Quality Characteristics of Pork Patties Containing Antioxidative Fish Skin Peptide or Fish Skin Peptide-loaded Nanoliposomes during Refrigerated Storage. Korean J. Food Sci. Anim. Resour..

[3] Sayas-Barbera E., Martin-Sanchez A.M., Cherif S., Ben-Abda J., Perez-Alvarez J.A. (2020). Effect of date (*Phoenix dactylifera L*.) pits on the shelf life of beef burgers. Foods.

[4] Guyon C., Meynier A., de Lamballerie M. (2016). Protein and lipid oxidation in meat: A review with emphasis on high pressure treatments. Trends Food Sci. Technol..

[5] Mariutti L.R.B., Bragagnolo N. (2017). Influence of salt on lipid oxidation in meat and seafood products: A review. Food Res. Int..

[6] Hartmann R.M., Martins M.I.M., Tieppo J., Fillmann H.S., Marroni N.P. (2012). Effect of *Boswellia serrata* on antioxidant status in an experimental model of colitis rats induced by acetic acid. Dig. Dis. Sci..

[7] Bae I.K., Kim K.J., Choi J.S., Choi Y.I., Ha J.H. (2019). Quality properties and storage characteristics of pyeonyuk with different additional levels of turmeric powder. Food Sci. Anim. Resour..

[8] Lorenzo J.M., Gonzalez-Rodriguez R.M., Sanchez M., Amado I.R., Franco D. (2013). Effects of natural (grape seed and chestnut extract) and synthetic antioxidants (buthylatedhydroxytoluene, BHT) on the physical, chemical, microbiological and sensory characteristics of dry cured sausage “chorizo”. Food Res. Int..

[9] Akwetey W.Y., Yamoah G. (2013). Producing low-fat pork patties with solar-dried plantain (*Musa Acuminate*) flour. J. Anim. Sci. Adv..

[10] Gao X.Q., Zhang W.G., Zhou G.H. (2014). Emulsion stability, thermo-rheology and quality characteristics of ground pork patties prepared with soy protein isolate and carrageenan. J. Sci. Food Agric..

[11] Andic S., Zorba O., Tuncturk Y. (2010). Effect of whey powder, skim milk powder and their combination on yield and textural properties of meat patties. Int. J. Agric. Biol..

[12] Iram F., Khan S.A., Husain A. (2017). Phytochemistry and potential therapeutic actions of Boswellic acids: A mini-review. Asian Pac. J. Trop. Biomed..

[13] Singh P., Chacko K.M., Aggarwal M.L., Bhat B., Khandal R.K., Sultana S., Kuruvilla B.T. (2012). A-90 day gavage safety assessment of *Boswellia serrata* in rats. Toxicol. Int..

[14] Siddiqui M.Z. (2011). Boswellia serrata, a potential antiinflammatory agent: An overview. Indian J. Pharm. Sci..

[15] Huang M.T., Badmaev V., Ding Y., Liu Y., Xie J.G., Ho C.T. (2000). Anti-tumor and anti-carcinogenic activities of triterpenoid, beta-boswellic acid. Biofactors.

[16] Beghelli D., Isani G., Roncada P., Andreani G., Bistoni O., Bertocchi M., Lupidi G., Alunno A. (2017). Antioxidant and ex vivo immune system regulatory properties of Boswellia serrata extracts. Oxid. Med. Cell Longev..

[17] Kiczorowska B., Samolinska W., AI-Yasiry A.R.M., Kowalczyk-Pecka D. (2016). Effect of *Boswellia serrata* dietary supplementation on growth performance, gastrointestinal microflora, and morphology of broilers. Ann. Anim. Sci..

[18] Catanzaro D., Rancan S., Orso G., Dall’Acqua S., Brun P., Giron M.C., Carrara M., Castagliuolo I., Ragazzi E., Caparrotta L. (2015). Boswellia serrata preserves intestinal epithelial barrier from oxidative and onflammatory damage. PLoS ONE.

[19] Smithers G.W. (2008). Whey and whey proteins-From ‘gutter-to-gold’. Int. Dairy J..

[20] Dziuba B., Dziuba M. (2014). Milk proteins-derived bioactive peptides in dairy products: Molecular, biological and methodological aspects. Acta Sci. Pol. Technol. Aliment..

[21] Bilyk O., Slyvka N., Gutyj B., Dronyk H., Sukhorska O. (2017). Substantiation of the method of protein extraction from sheep and cow whey for producing the cheese “Urda”. East. Eur. J. Enterp. Technol..

[22] Devries M.C., Phillips S.M. (2015). Supplemental protein in support of muscle mass and health: Advantage whey. J. Food Sci..

[23] Patel S. (2015). Functional food relevance of whey protein: A review of recent findings and scopes ahead. J. Funct. Food.

[24] Zouari N., Ayadi M.A., Hadj-Taieb S., Frikha F., Attia H. (2012). Whey powder, ι-carrageenan, and fat interactions and their influence on instrumental texture and sensory properties of turkey meat sausage using a mixture design approach. Int. J. Food Prop..

[25] Haak L., Raes K., De Smet S. (2009). Effect of plant phenolics, tocopherol and ascorbic acid on oxidative stability of pork patties. J. Sci. Food Agric..

[26] Jridi M., Siala R., Fakhfakh N., Ayadi M.A., Elhatmi M., Taktak M.A., Nasri M., Zouari N. (2015). Effect of rosemary leaves and essential oil on turkey sausage quality. Acta Aliment..

[27] Kim H.S., Chin K.B. (2016). Effects of Drying Temperature on Antioxidant Activities of Tomato Powder and Storage Stability of Pork Patties. Korean J. Food Sci. Anim Resour..

[28] Association of Official Analytical Chemists (2012). Official Methods of Analysis.

[29] Murphy E.W., Criner P.E., Grey B.C. (1975). Comparison of methods for calculation retention of nutrients in cooked foods. J. Agric. Food Chem..

[30] Buege J.A., Aust S.D. (1978). Microsomal Lipid Peroxidation. Methods in Enzymology.

[31] Conway E.J. (1962). Determination of volatile amines. Microdiffusion Analysis and Volumetric Error.

[32] Serdaroglu M. (2006). Improving low fat meatball characteristics by adding whey powder. Meat Sci..

[33] Ha J.H., Lee J.H., Lee J.J., Choi Y.I., Lee H.J. (2019). Effects of whey protein injection as a curing solution on chicken breast meat. Food Sci. Anim. Resour..

[34] Taylor B.J., Walsh M.K. (2006). Development and sensory analysis of a textured whey protein meatless patty. Food Sci..

[35] El-Magoli S., Larola S., Hansen P.M.T. (1995). Ultrastructure of low-fat ground beef patties with added whey protein concentrate. Food Hydrocoll..

[36] Brewer M.S., Novakofski J., Freise K. (2006). Instrumental evaluation of pH effects on ability of pork chops to bloom. Meat Sci..

[37] Abdelmalek Y.B., Essid I., Smeti S., Atti N. (2018). The anti-oxidant and antimicrobial effect of Rosmarinus officinalis L. distillation residues’ intake on cooked sausages from ewes fed linseed. Small Rumin. Res..

[38] Choi Y.S., Choi J.H., Han D.J., Kim H.Y., Kim H.W., Lee M.A., Chung H.J., Kim C.J. (2012). Effects of Laminaria japonica on the physico-chemical and sensory characteristics of reduced-fat pork patties. Meat Sci..

[39] Holman B.W.B., Ponnampalam E.N., van de Ven R.J., Kerr M.G., Hopkins D.L. (2015). Lambmeat colour values (HunterLab CIE and reflectance) are influenced by aperture size (5 mm v. 25 mm). Meat Sci..

[40] Faustman C., Sun Q., Mancini R., Suman S.P. (2010). Myoglobin and lipid oxidation interactions: Mechanistic bases and control. Meat Sci..

[41] Atughonu A.G., Zayas J.F., Herald T.J., Harbers L.H. (1998). Thermo-rheology, quality characteristics, and microstructure of frankfurters prepared with selected plant and milk additives. J. Food Qual..

[42] Zhang X., Xu Y., Xue H., Jiang G.C., Liu X.J. (2019). Antioxidant activity of vine tea (Ampelopsis grossedentata) extract on lipid and protein oxidation in cooked mixed pork patties during refrigerated storage. Food Sci. Nutr..

[43] Ozer O., Saricoban C., Unal K. (2018). The Effects of phytic acid, carnosine and butylated hydroxylanisole on some properties of mechanically deboned chicken patties during frozen storage. Selcuk J. Agric. Food Sci..

[44] Wan Rosli W.I., Solihah M.A., Aishah M., Nik Fakurudin N.A., Mohsin S.S.J. (2011). Colour, textural properties, cooking characteristics and fibre content of chicken patty added with oyster mushroom (*Pleurotus sajor-caju*). Int. Food Res. J..

[45] Verma A.K., Chatli M.K., Kumar D., Kumar P., Mehta N. (2015). Efficacy of sweet potato powder and added water as fat replacer on the quality attributes of low-fat pork patties. Asian Australas. J. Anim. Sci..

[46] Gray J.I., Gomaa E.A., Buckley D.J. (1996). Oxidative quality and shelf life of meats. Meat Sci..

[47] Niebler J., Buettner A. (2015). Identification of odorants in frankincense (*Boswellia sacra* Flueck.) by aroma extract dilution analysis and two-dimensional gas chromatography-mass spectrometry/olfactometry. Phytochemistry.

[48] Wojciak K.M., Dolatowski Z.J. (2016). Shelf life of organic roast pork enriched with acid whey-plant extracts combination. J. Food Qual..

[49] Schoina V., Terpou A., Papadaki A., Bosnea L., Kopsahelis N., Kanellaki M. (2020). Enhanced aromatic profile and functionality of cheese whey beverages by incorporation of probiotic cells immobilized on pistacia terebinthus resin. Foods.

[50] Legrand D. (2012). Lactoferrin, a key molecule in immune and inflammatory processes. Biochem. Cell Biol..

